# Humoral and Cellular Immune Response After a 3-Dose Heterologous SARS-CoV-2 Vaccination Using the mRNA-BNT162b2 and Viral Vector Ad26COVS1 Vaccine in Hemodialysis Patients

**DOI:** 10.3389/fimmu.2022.907615

**Published:** 2022-06-23

**Authors:** Tamara Davidovic, Judith Schimpf, Armin Abbassi-Nik, Richard Stockinger, Hannelore Sprenger-Mähr, Karl Lhotta, Emanuel Zitt

**Affiliations:** ^1^ Department of Internal Medicine 3 (Nephrology and Dialysis), Feldkirch Academic Teaching Hospital, Feldkirch, Austria; ^2^ Institute of Pathology, Feldkirch Academic Teaching Hospital, Feldkirch, Austria; ^3^ Vorarlberg Institute for Vascular Investigation and Treatment (VIVIT), Feldkirch, Austria; ^4^ Agency for Preventive and Social Medicine (aks), Bregenz, Austria

**Keywords:** Ad26COVS1, COVID-19, hemodialysis, BNT162b2, SARS-CoV-2, vaccination

## Abstract

**Background:**

Due to the waning humoral response after a two-dose SARS-CoV-2 mRNA vaccination, a third booster was recommended in hemodialyis patients. Data on a heterologous mRNA-vector regimen, which might improve immunogenicity, are very limited.

**Methods:**

In this observational study 36 chronic hemodialysis patients (mean (SD) age 66.9 (15.9) years, 33.3% females) were followed up for 13 months. All patients were vaccinated twice using the mRNA-BNT162b2 vaccine, followed by a 3^rd^ dose of the vector vaccine Ad26COVS1 eight months later. We assessed the humoral response by quantifying the anti-SARS-CoV-2 spike IgG antibody and neutralizing antibody concentrations. The cellular immune response was evaluated *via* SARS-CoV-2 spike protein-specific interferon-γ release assay.

**Results:**

The seroconversion rate was 47.2%, 100%, 69.4% and 100% one month after the 1^st^ dose, one and six months after the 2^nd^ dose and four months after the heterologous 3^rd^ dose. The median (Q1, Q3) anti-SARS-CoV-2 spike IgG concentrations at the same time were 28.7 (13.2, 69.4) BAU/ml, 1130.0 (594.5, 1735.0) BAU/ml, 89.7 (26.4, 203.8) BAU/ml, and 2080.0 (1062.5, 2080.0) BAU/ml. The percentage of patients with neutralizing antibodies was 58.3% after the 2^nd^ dose and improved to 100% after the 3^rd^ dose (*P <*0.001). A positive T-cell response was found in 50% of patients after the 3^rd^ dose.

**Conclusions:**

A third heterologous booster dose helped to sustain humoral immunity in almost all hemodialysis patients and induced a significant T-cellular response in half of them. Stimulating the immune response against SARS-CoV-2 by two different vaccine platforms seems to be a promising approach.

## Introduction

Numerous reports about the short- and mid-term immune response up to 6 months after SARS-CoV-2 vaccination in hemodialysis patients have been published showing a seroconversion rate of up to 98% after a regular 2-dose mRNA vaccination with a significantly waning humoral response thereafter dependent on the vaccine type (1, 2). Hemodialysis patients show a high disease burden and have been found to be at highest risk for a severe course and death caused by SARS-CoV-2 infection (3–5). Therefore, a third booster dose has been recommended to enhance the protective immune response in this vulnerable patient cohort, and first short-term observations after homologous triple vaccination have been reported (6–9). A heterologous mRNA-vector vaccine regimen might improve immunogenicity outcomes and cellular response (10). However, to our knowledge there are no serial long-term data available including the vaccine-induced humoral and cellular immune response after a heterologous triple vaccination regimen including the mRNA vaccine BNT162b2 and vector vaccine Ad26COVS1. Therefore, we aimed to study the antibody and T-cell response during a follow-up of 13 months in chronic hemodialysis patients. We have already reported safety, tolerability, short-term and 6-months immunogenicity data for a larger cohort including these patients after prime mRNA vaccination in detail elsewhere (1, 11).

## Patients and Methods

### Study Population

In January 2021, all chronic in-center hemodialysis patients treated at Feldkirch Academic Teaching Hospital (n=87), Austria, were invited to receive the SARS-CoV-2 vaccination following the prioritization by the National Vaccination Committee. Only patients with a negative SARS-CoV-2 infection history and negative anti SARS-CoV-2-serology were included in accordance with the National Vaccination Recommendation. Thirty-seven patients were excluded initially, because they had declined vaccination (n=28), had recovered from Covid-19 (n=8) or were pregnant (n=1). After the 1^st^ vaccine dose (n=50) two patients were excluded due to death after dialysis withdrawal (n=1) and symptomatic SARS-CoV-2 infection (n=1). After the 2^nd^ dose (n=48) one patient died due to acute heart failure and was excluded. For the evaluation 6 months after the 2^nd^ dose (n=41) six patients had to be excluded due to successful kidney transplantation (n=1), death (n=4) and temporary stay abroad (n=1). For the final analysis (n=36) five additional patients had to be excluded due to death (n=1), successful kidney transplantation (n=1), missing 3^rd^ vaccine dose (n=1) and homologous 3^rd^ mRNA vaccination (n=2).

### Vaccination Periods

**Figure 1:**
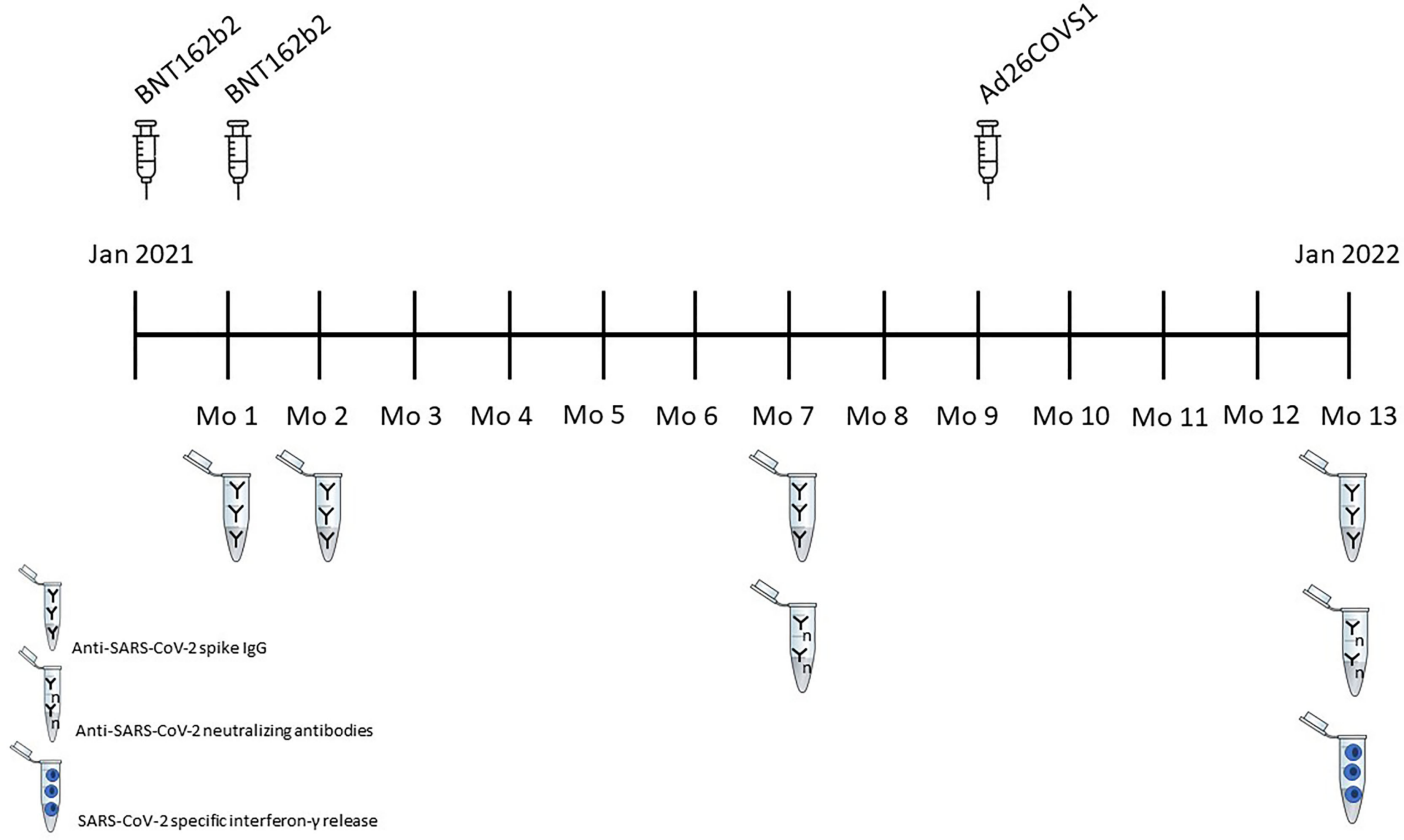
Vaccination Flow diagram.

After written informed consent all patients were vaccinated with the Pfizer/BioNTech mRNA-BNT162b2 SARS-CoV-2 vaccine (COMIRNATY^®^, 30 µg). This lipid nanoparticle–formulated, nucleoside-modified RNA vaccine encodes the SARS-CoV-2 full-length spike protein, modified by two proline mutations to lock it in the prefusion conformation (12, 13). The first doses were given on January 9^th^ and 11^th^, the second doses on February 4^th^ and 5^th^ 2021 with a 4-week interval. For the third booster dose we used the Janssen viral vector Ad26COVS1vaccine (500 µl), which was given 8 months later, on September 29^th^ and 30^th^. This is a recombinant, replication-incompetent adenovirus serotype 26 vector encoding a full-length and stabilized SARS-CoV-2 spike protein (14, 15). A vaccination flow diagram is presented in **Figure 1**.

### Anti-SARS-CoV-2 Immunoassays

Antibody response was determined after the first dose on the day of the second dose (month 1), 28 days (month 2) and again 180 days (month 7) after the second vaccine dose, as well as 120 days (month 13) after the third heterologous dose by quantifying IgG antibodies from the patients´ serum. We used the LIAISON^®^ SARS-CoV-2-TrimericS IgG chemiluminescent immunoassay (Diasorin S.p.A., Saluggia, Italy). The assay detects IgG antibodies against recombinant trimeric spike glycoprotein including the receptor-binding domain (RBD) and the N-terminal domain sites from the S1 subunit. The assay has a clinical sensitivity of 98.7%, a specificity of 99.5% (95% confidence interval [95% CI] 99.0% - 99.7%) and has a very good correlation with the microneutralization test with a positive predictive agreement of 100% (95% CI 97.8% - 100%) and a negative predictive agreement of 96.9% (95% CI 92.9% - 98.7%). Test results were adapted to the WHO International standard for anti-SARS-CoV-2 immunoglobulin binding activity and presented in binding activity units per milliliter (BAU/ml) following the conversion equation “AU/ml*2.6 = BAU/ml” according to the manufacturer. A value of ≥33.8 BAU/ml was considered as evidence of a positive vaccination antibody response with seroconversion. The assay range according to the manufacturer is 4.81 - 2080 BAU/ml.

At month 7 and 13, we also determined anti-SARS-CoV-2 nucleocapsid antibodies to exclude an asymptomatic natural SARS-CoV-2 infection during or after the vaccination interval. We used the Platelia^®^ SARS-CoV-2 Total Ab ELISA (BIO-RAD, Marnes-la-Coquette, France) for the detection of total anti-SARS-CoV-2 nucleocapsid antibodies (IgM/IgA/IgG). The assay uses a recombinant SARS nucleocapsid protein in a one-step antigen capture format assay. The presence of anti-SARS-CoV-2 nucleocapsid antibodies in an individual specimen is determined by comparing the optical density reading of the specimen to the optical density of the cut-off control. A specimen ratio <0.8 is considered to be negative for the presence of anti-SARS-CoV-2 nucelocapsid antibodies. The assay has a clinical sensitivity of 100% (95% CI 98.7 - 99.9%), a specificity of 99.5% (95% CI 98.6% - 99.8%), and a positive predictive agreement with PCR positivity of 100% (95% CI 98.7% - 99.9%) and a negative predictive agreement of 99.5% (95% CI 98.6% - 99.8%).

### Neutralization Assay

Additionally, neutralizing antibodies were assessed using the cPass™ SARS-CoV-2 Surrogate Virus Neutralization Test (SVNT) assay (GenScript, Piscataway Township, USA), according to the manufacturer’s specifications. Briefly, the serum is incubated with horseradish peroxidase (HRP)-conjugated spike-RBD and transferred to ACE2 coated wells. Neutralizing antibodies present in serum will bind to HRP-RBD inhibiting RBD-ACE2 interactions and thus are removed during the following washing steps. Only unbound HRP-RBD as well as HRP-RBD bound to non-neutralizing antibodies will be captured by ACE2 on the plate. The following absorbance measurement is inversely dependent on the titer of anti-SARS-CoV-2 neutralizing antibodies. A 30% signal inhibition was used as cutoff, with <30% classified as negative; 30-100% signal inhibition representing a range of low-to-high neutralization ability. The negative and positive percent agreement with conventional plaque reduction neutralization test (PRNT)_50_ and PRNT_90_ assays is approximately 100%. The sensitivity and specificity for the assay is 93.80% and 99.4%, respectively.

### Interferon-γ Release Assay

To determine the cellular immune response, we used the QuantiFERON^®^ SARS-CoV-2 assay (Qiagen, Germantown, USA), a SARS-CoV-2 spike protein-specific interferon-γ release assay (IGRA). The assay uses specialized blood collection tubes including two antigen tubes, SARS-CoV-2 Ag1 and SARS-CoV-2 Ag2, comprising a combination of antigens specific to SARS-CoV-2 to stimulate CD4+- and CD8+- T-lymphocytes in heparinized whole blood. Blood was collected prior to the initiation of the dialysis session. The tubes were incubated as soon as possible, and within 16 hours of collection at 37°C ± 1°C for 16 to 24 hours. In the plasma from the stimulated samples interferon-γ was detected using the QuantiFERON^®^ SARS-CoV-2 ELISA according to the manufacturer’s specifications. Interferon-γ values (IU/ml) for Ag1, Ag2 and the Mitogen control were corrected for background by subtracting the value for the Nil control. According to the manufacturer interferon-γ concentrations >0.15 IU/ml for one or both antigens indicated positivity. With this cut-off value the assay has a sensitivity of 98.3% (95% CI: 91.1% - 100%) and a specificity of 100% (95% CI: 94.0% - 100%). The lower limit of detection of the ELISA is 0.065 IU/ml.

### Ethical Considerations

The study was conducted in compliance with the Helsinki Declaration of 1975, as revised in 2013, and Good Clinical Practices. The study protocol was approved by the institutional review board and the ethics committee of Innsbruck Medical University.

### Statistical Analyses

Categorical data are presented as absolute and relative number of patients. For continuous data mean and standard deviation (SD) or median with interquartile range (1^st^ quartile, 3^rd^ quartile) was used, depending on its distribution. Repeated measures ANOVA or Friedman test was used for comparisons applying a Bonferroni correction to multiple testing for pairwise comparisons, categorical parameters were compared using the Chi-squared or Fisher´s exact tests. A two-sided *P* value < 0.05 was deemed to indicate statistical significance. All statistical analyses were performed with IBM SPSS Statistics 26 (IBM, Armonk (NY), USA).

## Results

Baseline characteristics of the 36 hemodialysis patients (mean (SD) age 66.9 (15.9) years, 33.3% females) with complete triple-vaccination and follow-up over the 13 months are given in **Table 1**.

**Table 1:** Baseline patients´ characteristics.

Characteristic	
Gender, n (%)	
Female	12 (33.3)
Male	24 (66.7)
Age (years), mean (SD)	66.9 (15.9)
Dialysis vintage (months), median (Q1, Q3)	35.0 (17.3, 62.0)
Renal disease, n (%)	
Hypertensive kidney disease	12 (33.3)
Diabetic kidney disease	5 (13.9)
Glomerulonephritis	8 (22.2)
Other	11 (30.6)
Vascular access	
Arteriovenous fistula, n (%)	25 (69.4)
Arteriovenous graft, n (%)	2 (5.6)
Central venous catheter, n (%)	9 (25.0)
spKt/V^#^, mean (SD)	1.54 (0.25)
Diabetes mellitus, n (%)	9 (25.0)
Albumin (g/dL), mean (SD)	4.0 (0.5)
CRP (mg/dL), mean (SD)	0.8 (0.9)
Hemoglobin (g/dL), mean (SD)	11.6 (1.3)
Calcium (mmol/L), mean (SD)	2.10 (0.17)
Phosphorus (mmol/L), mean (SD)	1.88 (0.47)
PTH (pg/mL), mean (SD)	300 (139)
25(OH)vitamin D (µg/L), mean (SD)	18.5 (12.6)
Calcitriol supplementation, n (%)	26 (72.2)
Prior kidney transplantation, n (%)	5 (13.9)
Immunosuppressive medication, n (%)	7 (19.4)
Glucocorticoid, n (%)	6 (16.7)
Azathioprine, n (%)	1 (2.8)
Hepatitis B vaccination seroconversion^*^, n (% of vaccinated)	17 (65.4)
Anti-HBs antibody concentration (IU/L), median (Q1, Q3)	111 (0.0, 352)

spKt/V, single-pool Kt/V; CRP, C-reactive protein; PTH, parathyroid hormone.

^#^spKt/V given as the weekly mean in the week prior to vaccination.

^*^Hepatitis B vaccination seroconversion defined by an anti-HBs antibody concentration ≥10 IU/L; n=10 patients with documented immunity after prior infection (positive anti-HBs and anti-HBc antibodies).

**Figure 2:**
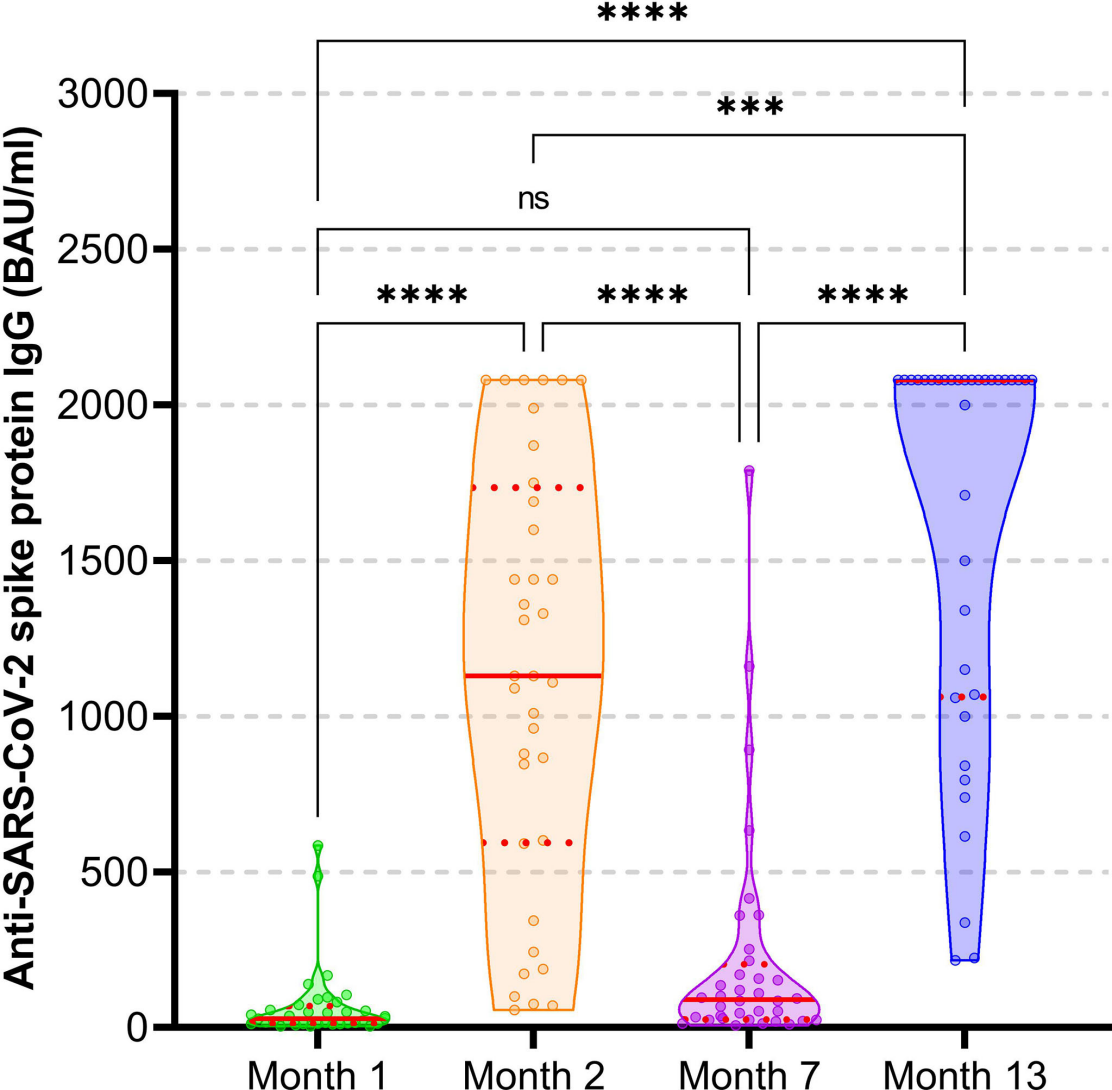
Anti-SARS-CoV-2-spike protein IgG concentration after heterologous triple vaccination with the mRNA-BNT162b2 and vector Ad26COVS1 vaccine in hemodialysis patients. Violin plots (combining box and kernel density plots) including individual data points are displayed. The red line indicates the median, the red dotted lines the first and third quartile. The threshold for seropositivity is ≥33.8 BAU/ml. ****P < 0.0001; ***P < 0.001; ns, not significant (*P* = 0.089).

Compared to the seroconversion (SC) rate of 47.2% and a median (Q1, Q3) anti-SARS-CoV-2 spike IgG concentration of 28.7 (13.2, 69.4) BAU/ml one month after the first vaccine dose, SC increased to 100% with a median anti-spike IgG concentration of 1130.0 (594.5, 1735.0) BAU/ml one month after the second dose. Six months later the SC rate decreased to 69.4% with a median anti-spike IgG concentration of 89.7 (26.4, 203.8) BAU/ml, but increased again to 100% with 2080.0 (1062.5, 2080.0) BAU/ml four months after the heterologous 3^rd^ dose (**Figure 2**). Except the comparison between month 1 and 7 (*P* = 0.089), anti-spike IgG concentrations differed significantly comparing the four time points (*P* < 0.001 for all).

To further analyze the neutralizing capacity, we additionally assessed neutralizing antibodies six months after complete mRNA vaccination and again four months after the heterologous vaccine Ad26COVS1. The median (Q1, Q3) percent virus neutralization was 40.4% (32.6, 47.1) at month 7 and significantly increased to 97.1% (89.8, 97.6) at month 13 (*P* < 0.001); the percentage of patients above the 30% threshold for neutralizing antibody positivity was 58.3% and 100% (*P* < 0.001), respectively.

With a specimen ratio cut-off value of <0.8, all patients had negative anti-SARS-CoV-2 nucleocapsid antibodies with a mean (SD) specimen ratio of 0.12 (0.04) at month 7 and 0.20 (0.14) at month 13, indicating a very low probability of an undetected asymptomatic natural infection between 2^nd^ and 3^rd^ and after the 3^rd^ vaccination.

A positive SARS-CoV-2 specific T-cell response assessed by IGRA was found in 50% of patients four months after the 3^rd^ vaccination (month 13), with a median (Q1, Q3) of 0.152 IU/ml (0.065, 1.373). A positive cellular response at month 13 was associated with higher anti-SARS-CoV-2 spike IgG concentrations at all four time points (month 1, month 2, month 7, month 13), although the difference between patients with and without positive T-cellular response reached statistical significance at month 1 only (month 1: 55.4 [21.7, 99.6] vs 15.5 [11.7, 38.6] BAU/ml, *P* = 0.004; month 2: 1440 [871.3, 2080] vs 995 [305, 1380] BAU/ml, *P* = 0.064; month 7: 128.5 [36.4, 469.8] vs 53 [20.8, 125.8] BAU/ml, *P* = 0.051; month 13: 2080 [1787.5, 2080] vs 1605 [781.8, 2080] BAU/ml, *P* = 0.126). Baseline characteristics from patients with a positive cellular response did not significantly differ from patients without cellular response, except that positive patients were more often treated with calcitriol (89% vs 56%, *P* = 0.026).

Overall, the heterologous 3^rd^ dose was well tolerated. Mild pain at the injection site was the only patient self-reported local reaction in a minority of patients. One patient with IgA nephropathy as primary renal disease reported about a vaccine-associated IgA nephropathy flare with gross hematuria for several days after the 3^rd^ dose, but without other systemic reactions. During the complete follow-up no patient acquired symptomatic and PCR-confirmed SARS-CoV-2 infection.

## Discussion

In our study we found a significantly improved immunogenicity including the neutralizing antibody response up to four months after a third heterologous SARS-CoV-2 vaccine dose in hemodialysis patients. Albeit a 2-months shorter follow-up, the SC rate four months after the 3^rd^ heterologous dose was significantly higher than six months after two homologous doses, with even higher absolute antibody concentrations compared to the evaluation one month after the homologous doses. This promising humoral response might indicate the possibility to extend the interval for future booster vaccinations in our extremely vulnerable hemodialysis patients.

After a high seroconversion rate following the two mRNA-vaccine doses, we observed a significant decline of anti-SARS-CoV-2 spike IgG concentrations over six months. This finding is in line with other reports (16). The early and fast waning of the humoral response prompted a third booster vaccination. Available data are very limited to assess the value of a heterologous compared to a homologous triple vaccination regimen in hemodialysis patients. No randomized controlled trials have investigated this question so far. In a recent observational study, Meijers et al. found significantly higher SARS-CoV-2 anti-S IgG concentrations in hemodialysis patients with triple homologous vaccination using the mRNA-1273 vaccine compared to a heterologous regimen based on 2 doses of the adenovirus-vector vaccine AZD1222 followed by BNT162b2 (17). No differences were found between the homologous triple BNT162b2 and the heterologous 2-dose AZD1222/BNT162b2, as well as between the homologous triple BNT162b2 and mRNA-1273 vaccinations. The seroconversion rate after triple vaccination was comparable between the three groups (AZD1222/BNT162b2 95.8%, BNT162b2 98%, mRNA-1273 100%). This seroconversion rate is well in line with our results, but in contrast to our study neutralizing antibodies and the cellular immune response were not determined by Meijers et al. and can therefore not be compared with our findings.

Our study is the first to evaluate the vector vaccine Ad26COVS1 as a third booster dose following a mRNA-vaccine-based 2-dose prime immunization. A mixed vaccine platform strategy was proposed to possibly improve the vaccination response with an enhanced neutralizing antibody response against SARS-CoV-2 variants with the mRNA-based vaccine and a stronger spike-protein specific T-cellular response elicited by the viral vector-based vaccine (18, 19). In our small study using such a mixed approach we observed an excellent humoral response with neutralizing antibody positivity in 100% of the patients and a positive SARS-CoV-2 specific T-cell response in half of them. Although no generally applicable absolute anti-spike protein antibody concentration has been defined to demonstrate clinically relevant seroprotection, a level >264 BAU/ml (95% CI: 108, 806) has been found to be associated with 80% vaccine efficacy against primary symptomatic Covid-19, although limited to the B.1.177 and B.1.1.7 SARS-CoV-2 variant (20). Ninety-four percent (34/36) of our patients with a third heterologous dose achieved this threshold. Whether this threshold indicates the same vaccine efficacy against the now dominant SARS-CoV-2 variant of concern B.1.1.529 Omicron is unknown. It remains to be proven whether a heterologous 3-dose regimen combining mRNA and vector vaccine improves the neutralizing humoral response against the now dominant Omicron variant and future emerging variants of concern in hemodialysis patients as has been shown in the general population (21) or enhances the variant-specific cellular immune response (22) which might translate into better clinical outcomes. A first report indicates the need of at least three vaccine doses to generate Omicron neutralizing antibodies in hemodialysis patients (23).

Our study results are limited by the low patient number and missing cellular response data in the early phase of our study. Nevertheless, the stringent follow-up of one and the same patients over 13 months with vaccination and response evaluation at always the same time in all patients allows a direct comparison without confounding and varying patients characteristics. All patients were serologically proven SARS-CoV-2 negative prior to vaccination. Therefore, we can exclude a booster effect of vaccination after undetected asymptomatic infection prior to the first vaccination course. Additionally, we assessed anti-SARS-CoV-2 nucleocapsid antibodies at month 7 and 13, which were negative for all patients at both times. Keeping in mind a potentially diminished serological response in our patients, these findings indicate a very low probability of an undetected asymptomatic natural infection amplifying the vaccine response, although not definitely excluding it. We did not perform regular PCR-based screening of asymptomatic patients during the follow-up. However, whenever a patient presented with equivocal symptoms, antigen- and PCR-based testing was applied. Due to the uniform ethnic nature of our Caucasian cohort, we cannot generalize our findings to other ethnicities. Last, our results are limited to SARS-CoV-2 infection-naïve seronegative patients and the heterologous vaccination schedule combining the 2-dose mRNA-BNT162b2 vaccine followed by Ad26COVS1.

## Conclusion

In conclusion, a third heterologous booster dose helped to sustain a protective humoral immunity in almost all patients of this extremely vulnerable cohort and induced a significant SARS-CoV-2 specific T-cellular response in half of them. Whether a heterologous vaccination regimen is superior to a homologous one in hemodialysis patients remains to be proven. Stimulating the immune response against SARS-CoV-2 by two different vaccine platforms is at least an attractive choice.

## Data Availability Statement

The original contributions presented in the study are included in the article/supplementary material. Further inquiries can be directed to the corresponding author.

## Ethics Statement

The studies involving human participants were reviewed and approved by Ethics Committee of Innsbruck Medical University. The patients/participants provided their written informed consent to participate in this study.

## Author Contributions

KL designed the study and revised the article; JS, AA-N, RS, and HS-M collected and interpreted data and revised the article; TD and EZ designed the study, analyzed and interpreted data, drafted and revised the article. All authors finally approved the submitted version of the article.

## Conflict of Interest

The authors declare that the research was conducted in the absence of any commercial or financial relationships that could be construed as a potential conflict of interest.

## Publisher’s Note

All claims expressed in this article are solely those of the authors and do not necessarily represent those of their affiliated organizations, or those of the publisher, the editors and the reviewers. Any product that may be evaluated in this article, or claim that may be made by its manufacturer, is not guaranteed or endorsed by the publisher.

## References

[1] Davidovic T Schimpf J Abbassi-Nik A Stockinger R Sprenger-Mahr H Lhotta K. Waning Humoral Response 6 Months After SARS-CoV-2 Vaccination With the mRNA-BNT162b2 Vaccine in Hemodialysis Patients: Time for a Boost. Kidney Int (2021) 100(6):1334–5. doi: 10.1016/j.kint.2021.10.006 PMC8516134 34656642

[2] El Karoui K De Vriese AS. COVID-19 in Dialysis: Clinical Impact, Immune Response, Prevention, and Treatment. Kidney Int (2022) 101(5):883–94. doi: 10.1016/j.kint.2022.01.022 PMC8842412 35176326

[3] Hilbrands LB Duivenvoorden R Vart P Franssen CFM Hemmelder MH Jager KJ. COVID-19-Related Mortality in Kidney Transplant and Dialysis Patients: Results of the ERACODA Collaboration. Nephrol Dial Transpl (2020) 35(11):1973–83. doi: 10.1093/ndt/gfaa261 PMC7665620 33151337

[4] Jager KJ Kramer A Chesnaye NC Couchoud C Sanchez-Alvarez JE Garneata L. Results From the ERA-EDTA Registry Indicate a High Mortality Due to COVID-19 in Dialysis Patients and Kidney Transplant Recipients Across Europe. Kidney Int (2020) 98(6):1540–8. doi: 10.1016/j.kint.2020.09.006 PMC7560263 32979369

[5] Williamson EJ Walker AJ Bhaskaran K Bacon S Bates C Morton CE. Factors Associated With COVID-19-Related Death Using OpenSAFELY. Nature (2020) 584(7821):430–6. doi: 10.1038/s41586-020-2521-4 PMC7611074 32640463

[6] Bensouna I Caudwell V Kubab S Acquaviva S Pardon A Vittoz N. SARS-CoV-2 Antibody Response After a Third Dose of the BNT162b2 Vaccine in Patients Receiving Maintenance Hemodialysis or Peritoneal Dialysis. Am J Kidney Dis (2022) 79(2):185–92. doi: 10.1053/j.ajkd.2021.08.005 PMC8425695 34508833

[7] Ducloux D Colladant M Chabannes M Yannaraki M Courivaud C. Humoral Response After 3 Doses of the BNT162b2 mRNA COVID-19 Vaccine in Patients on Hemodialysis. Kidney Int (2021) 100(3):702–4. doi: 10.1016/j.kint.2021.06.025 PMC8243640 34216675

[8] Espi M Charmetant X Barba T Mathieu C Pelletier C Koppe L. A Prospective Observational Study for Justification, Safety, and Efficacy of a Third Dose of mRNA Vaccine in Patients Receiving Maintenance Hemodialysis. Kidney Int (2022) 101(2):390–402. doi: 10.1016/j.kint.2021.10.040 34856313 PMC8628628

[9] Robert T Lano G Giot M Fourie T de Lamballeri X Jehel O. Humoral Response After SARS-CoV-2 Vaccination in Patients Undergoing Maintenance Haemodialysis: Loss of Immunity, Third Dose and non-Responders. Nephrol Dial Transpl (2022) 37(2):390–2. doi: 10.1093/ndt/gfab299 34643714

[10] Deming ME Lyke KE. A 'Mix and Match' Approach to SARS-CoV-2 Vaccination. Nat Med (2021) 27(9):1510–1. doi: 10.1038/s41591-021-01463-x PMC12036724 34312555

[11] Zitt E Davidovic T Schimpf J Abbassi-Nik A Mutschlechner B Ulmer H. The Safety and Immunogenicity of the mRNA-BNT162b2 SARS-CoV-2 Vaccine in Hemodialysis Patients. Front Immunol (2021) 12:704773. doi: 10.3389/fimmu.2021.704773 34220867 PMC8242233

[12] Walsh EE Frenck RW Jr. Falsey AR Kitchin N Absalon J Gurtman A. Safety and Immunogenicity of Two RNA-Based Covid-19 Vaccine Candidates. N Engl J Med (2020) 383(25):2439–50. doi: 10.1056/NEJMoa2027906 PMC7583697 33053279

[13] Wrapp D Wang N Corbett KS Goldsmith JA Hsieh CL Abiona O. Cryo-EM Structure of the 2019-Ncov Spike in the Prefusion Conformation. Science (2020) 367(6483):1260–3. doi: 10.1126/science.abb2507 PMC7164637 32075877

[14] Bos R Rutten L van der Lubbe JEM Bakkers MJG Hardenberg G Wegmann F. Ad26 Vector-Based COVID-19 Vaccine Encoding a Prefusion-Stabilized SARS-CoV-2 Spike Immunogen Induces Potent Humoral and Cellular Immune Responses. NPJ Vaccines (2020) 5:91. doi: 10.1038/s41541-020-00243-x 33083026 PMC7522255

[15] Sadoff J Le Gars M Shukarev G Heerwegh D Truyers C de Groot AM. Interim Results of a Phase 1-2a Trial of Ad26.COV2.S Covid-19 Vaccine. N Engl J Med (2021) 384(19):1824–35. doi: 10.1056/NEJMoa2034201 PMC7821985 33440088

[16] Biedunkiewicz B Tylicki L Slizien W Lichodziejewska-Niemierko M Dabrowska M Kubanek A. Waning Humoral Response After COVID-19 mRNA Vaccination in Maintenance Dialysis Patients and Recovery After a Complementary Third Dose. Vaccines (Basel) (2022) 10(3):433. doi: 10.3390/vaccines10030433 35335065 PMC8950255

[17] Meijers B Goedgezelschap A Peeters D van der Veen A Verbinnen M Vermeersch P. Heterologous vs. Homologous Triple Anti-COVID-19 Vaccine Regimens in Patients on Maintenance Hemodialysis. Nephrol Dial Transpl (2022). doi: 10.1093/ndt/gfac033 PMC9383434 35138374

[18] Barros-Martins J Hammerschmidt SI Cossmann A Odak I Stankov MV Morillas Ramos G. Immune Responses Against SARS-CoV-2 Variants After Heterologous and Homologous ChAdOx1 Ncov-19/BNT162b2 Vaccination. Nat Med (2021) 27(9):1525–9. doi: 10.1038/s41591-021-01449-9 PMC8440184 34262158

[19] Schmidt T Klemis V Schub D Mihm J Hielscher F Marx S. Immunogenicity and Reactogenicity of Heterologous ChAdOx1 Ncov-19/mRNA Vaccination. Nat Med (2021) 27(9):1530–5. doi: 10.1038/s41591-021-01464-w PMC8440177 34312554

[20] Feng S Phillips DJ White T Sayal H Aley PK Bibi S. Correlates of Protection Against Symptomatic and Asymptomatic SARS-CoV-2 Infection. Nat Med (2021) 27(11):2032–40. doi: 10.1038/s41591-021-01540-1 PMC8604724 34588689

[21] Cheng SMS Mok CKP Leung YWY Ng SS Chan KCK Ko FW. Neutralizing Antibodies Against the SARS-CoV-2 Omicron Variant BA.1 Following Homologous and Heterologous CoronaVac or BNT162b2 Vaccination. Nat Med (2022) 28(3):486–9. doi: 10.1038/s41591-022-01704-7 PMC8940714 35051989

[22] Atmar RL Lyke KE Deming ME Jackson LA Branche AR El Sahly HM. Homologous and Heterologous Covid-19 Booster Vaccinations. N Engl J Med (2022) 386(11):1046–57. doi: 10.1056/NEJMoa2116414 PMC8820244 35081293

[23] Carr EJ Wu M Harvey R Billany RE Wall EC Kelly G. Omicron Neutralising Antibodies After COVID-19 Vaccination in Haemodialysis Patients. Lancet (2022) 399(10327):800–2. doi: 10.1016/S0140-6736(22)00104-0 PMC8776276 35065703

